# Draft Genome Sequences of Five Strains in the Genus *Thauera*

**DOI:** 10.1128/genomeA.00052-12

**Published:** 2013-01-31

**Authors:** Binbin Liu, Åsa Frostegård, James P. Shapleigh

**Affiliations:** a Department of Chemistry, Biotechnology and Food Science, Norwegian University of Life Sciences, Ås, Norway; b Department of Microbiology, Cornell University, Ithaca, New York, USA

## Abstract

*Thauera* species are members of the betaproteobacteria and are most noted for their ability to metabolize aromatic compounds under anoxic conditions. Here, we announce the draft genome sequences of five *Thauera* strains in an effort to provide further genetic information as a resource for understanding the ecological function of this environmentally important genus.

## GENOME ANNOUNCEMENT

The genus *Thauera* was proposed as a new genus in 1993 by Macy et al. (1) and was named after the German microbiologist Rudolf Thauer. This genus currently consists of nine named species and one recently announced novel species (2). Organisms within *Thauera* have been isolated from wastewater treatment plants, where they may be a dominant part of the microbial community (3–8). They are also found in other environments, such as soils and sediments (9, 10), and are known for their versatile metabolism. The first strain in this genus for which a completely sequenced genome was obtained was *Thauera aminoaromatica* MZ1T, which was isolated from activated sludge samples from an industrial wastewater treatment facility (7). The strain is noted for its ability to degrade various aromatic compounds and for production of abundant exopolysaccharide (11).

In this study, we sequenced the whole genomes of five *Thauera* strains: *T. aminoaromatica* S2, *T. linaloolentis* 47Lol, and *Thauera* spp. 27, 28, and 63 (Table 1). *T. aminoaromatica* S2 was found to be capable of degrading various aromatic substrates, including benzoate, phenylacetate, and *p*-cresol, under denitrifying conditions (4). *T. linaloolentis* 47Lol has been found to utilize aliphatic monoterpenes as the sole electron donor and carbon source under denitrifying conditions (10). *Thauera* sp. 27 and *Thauera* sp. 28 were isolated from a laboratory-scale denitrifying reactor fed with acetate and nitrate and seeded with methanogenic granules (12, 13). Our recent results based on a phenotypic comparison showed that strains in the genus *Thauera* can be divided into two distinct groups according to their denitrification-regulatory phenotype (DRP) characteristics (14); four strains were characterized by a rapid, complete onset (RCO) of expression of all denitrification genes, and one strain showed progressive onset (PO) of expression of denitrification genes (B. Liu and Å. Frostegård, unpublished data). To gain better insight into the genetic and biochemical mechanisms underlying the different denitrification phenotypes, whole-genome sequencing of these strains was undertaken.

**TABLE 1:** Summary of information for the whole genomes of five *Thauera* strains: *T. aminoaromatica* S2, *T. linaloolentis* 47Lol, and *Thauera* spp. 27, 28, and 63

Strain	Origin	Place	Phenotypic group	G+C content (%)*^a^*	Genome size (bp)^*b*^	No. of predicted features (PEGs+ RNAs)^*c*^	Accession no.	No. of contigs (>200 bp)	Reference(s)
*Thauera* sp. 27	Anaerobic sludge	Michigan	RCO	65.8	4,708,725	4,298	AMXB01000000	128	12, 13
*Thauera* sp. 28	Anaerobic sludge	Michigan	RCO	65.9	4,319,739	3,902	AMXA01000000	119	12, 13
*T. linaloolentis* 47Lol	Activated sludge	Germany	RCO	66.6	4,259,404	3,927	AMXE01000000	220	10
*Thauera* sp. 63	Anaerobic sludge	Michigan	RCO	66.2	4,183,036	3,942	AMXC01000000	103	12, 13
*T. aminoaromatica* S2	Anoxic ditch sludge	Constance, Germany	PO	68.6	4,252,780	3,897	AMXD01000000	317	4

^a^ The G+C content was determined with all the contigs of each strain using Artemis.

^b^ The genome size was estimated using either Velvet or CLC Genomics Workbench; the result corresponding to the greater number of features in the RAST pipeline is shown.

^c^ Features predicted using the RAST pipeline. A protein-encoding gene (PEG) is equivalent to a CDS (coding sequence).

Genomic DNA was extracted according to the method described previously (15). Whole-genome sequencing of five *Thauera* strains (Table 1) was performed using both paired-end and mate-pair reads on an Illumina HiSeq2000 instrument (Illumina, Inc., San Diego, CA) in the Norwegian Sequencing Centre (NSC; Oslo, Norway). The raw sequences were filtered by the FASTX-Toolkit (http://hannonlab.cshl.edu/fastx_toolkit/), and the quality of the reads was checked with the program FastQC (http://www.bioinformatics.babraham.ac.uk/projects/fastqc/). *De novo* assembly was performed using both Velvet version 1.2.07 (16) and CLC Genomics Workbench. Gene prediction and annotation were carried out using the RAST annotation server (17). The G+C content was calculated with the sequences of all the contigs of each strain using Artemis (18). Consistent with previous reports, the genome G+C contents of these strains were relatively high, ranging from 65.8% to 68.6%. Genome information for each strain is summarized in Table 1.

### Nucleotide sequence accession numbers. 

The draft genome sequences of the *Thauera* strains in this study have been deposited as whole-genome shotgun projects (BioProject ID no. PRJNA175409, PRJNA171225, PRJNA175412, PRJNA175413, and PRJNA175415) at DDBJ/EMBL/GenBank under the accession numbers AMXA00000000, AMXB00000000, AMXC00000000, AMXD00000000, and AMXE00000000. The versions described in this paper are the first versions, AMXA01000000, AMXB01000000, AMXC01000000, AMXD01000000, and AMXE01000000.
